# FRESH-bioprinted vascular constructs: a quantitatively characterized workflow for reproducible device-oriented applications

**DOI:** 10.3389/fbioe.2026.1920619

**Published:** 2026-09-30

**Authors:** Franca Scocozza, Elena Delgrosso, Michele Conti

**Affiliations:** 1 Department of Civil Engineering and Architecture, University of Pavia, Pavia, Italy; 2 3D and Computer Simulation Laboratory, IRCCS Policlinico San Donato, San Donato Milanese, Italy

**Keywords:** biofabrication, endovascular device testing, FRESH bioprinting, micro-CT, quantitative characterization, vascular construct

## Abstract

The lack of standardized and reproducible *in vitro* vascular models remains a major obstacle to the quantitative evaluation of endovascular devices and vessel–device interactions. While recent bioprinted vascular constructs have increased biological complexity, reproducibility, shape fidelity, and mechanical consistency remain insufficiently characterized. Here, we establish a quantitatively characterized FRESH-bioprinted workflow that enables reproducible fabrication and standardized characterization of vessel-like constructs for device-oriented testing. Bioink formulation, printing parameters, and post-processing conditions were systematically optimized. At the same time, construct geometry and mechanical reproducibility were quantitatively evaluated using micro-computed tomography (micro-CT), confined compression, and ring tensile testing. Sterile fabrication, preliminary cellularization, controlled perfusion, and intraluminal stent-like ring deployment were further explored to assess procedural compatibility within a device-oriented vascular testing framework. The optimized workflow enabled reproducible fabrication of vessel-like constructs across multiple geometrical scales, with close agreement between designed and measured dimensions and limited batch-to-batch variability. Micro-CT analysis revealed controlled post-processing-induced geometrical changes, including mild shrinkage and lumen reduction, while mechanical testing demonstrated reproducible axial and circumferential responses across independent fabrication batches. Intraluminal deployment of a radiopaque polycaprolactone ring induced local vessel expansion of approximately 8% while preserving lumen patency and structural integrity under low-pressure perfusion conditions. Preliminary biological assays confirmed high cell viability for up to 14 days and initial endothelial adhesion, supporting compatibility with sterile cell culture procedures and providing a robust foundation for future biologically advanced vascular models. By integrating biofabrication, quantitative imaging, mechanical characterization, and preliminary biological validation into a single workflow, this work establishes a quantitatively characterized and reproducible FRESH-bioprinted vascular platform that bridges the gap between rigid vascular phantoms and biologically advanced tissue-engineered systems. The proposed platform provides a robust experimental foundation for future process standardization, device-oriented vascular testing, and the progressive development of standardized experimental models for endovascular research.

## Graphical Abstract

**Figure:**
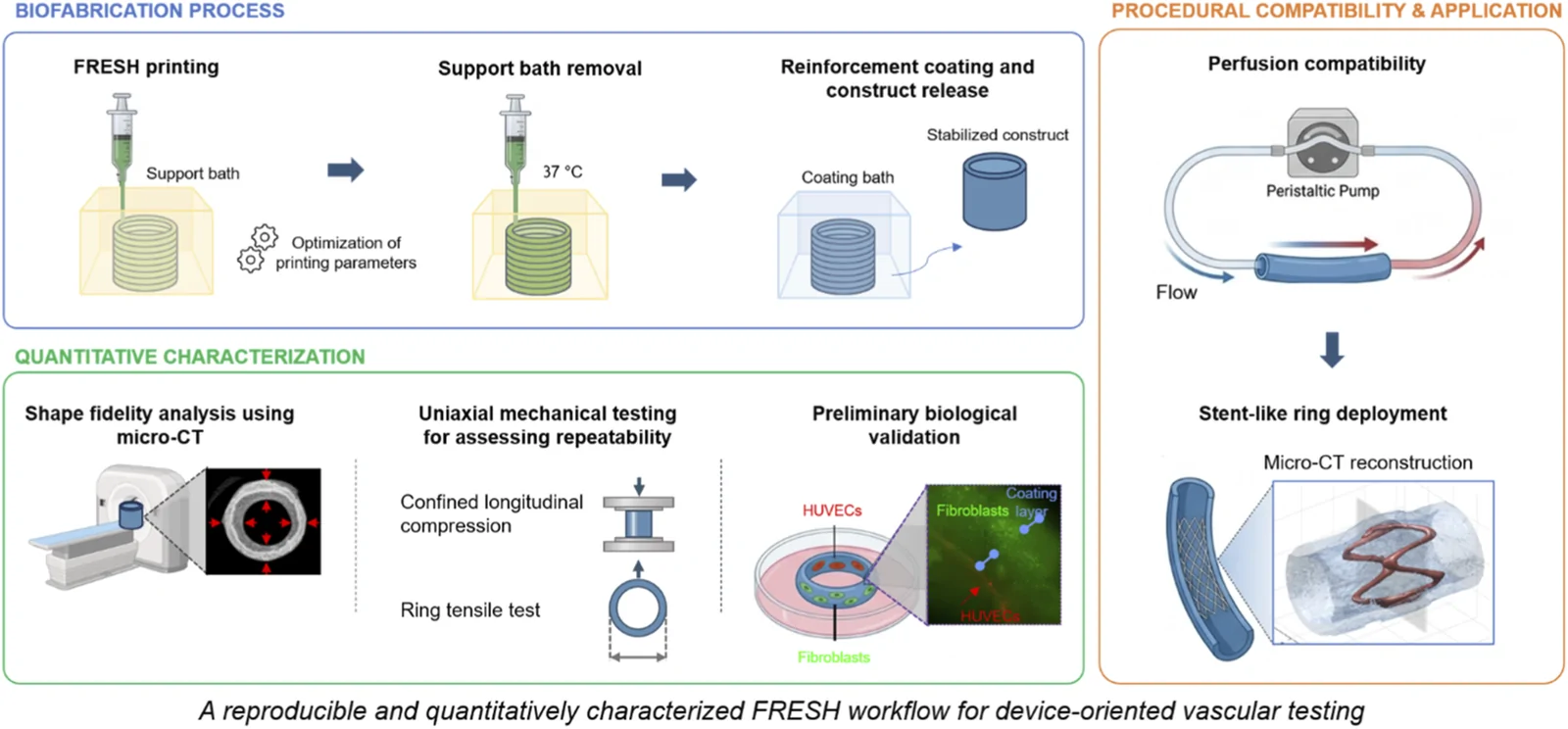

## 1 Introduction

Endovascular devices, such as stents and stent-grafts, are typically developed and validated through a combination of computational modeling, benchtop testing, and *in vivo* studies. Within this translational pipeline, *in vitro* vascular models serve as complementary experimental platforms to investigate device deployment, vessel–device interactions, imaging compatibility, and perfusion behavior under controlled conditions. Despite substantial technological progress, however, standardized and reproducible vascular models explicitly conceived as reference benchmarks for device-oriented evaluation remain limited (Sarode and Roy, 2019; Wisotzki et al., 2022). Consequently, existing *in vitro* platforms span a broad spectrum of complexity while rarely providing a shared quantitative and reproducible benchmarking framework.

Current vascular models range from rigid anatomical phantoms to biologically advanced tissue-engineered systems. Rigid or semi-rigid replicas fabricated from glass, silicone, acrylic, or 3D-printed polymers are widely employed for navigation, deployment testing, and flow visualization, particularly in neurovascular and aortic applications (Epshtein et al., 2025; Norris et al., 2022; Siewert et al., 2024; Johnson et al., 2022; Taneva et al., 2023). Although these systems provide excellent geometric fidelity and procedural robustness, they remain limited in reproducing realistic wall mechanics and soft-tissue deformation. Compliance-matched and biomimetic flow models partially address these limitations by incorporating tunable hardness, lubricity, and radial compliance, thereby enabling more realistic evaluation of vessel–device interactions and hemodynamics (Wisotzki et al., 2022; Norris et al., 2022; McCue et al., 2019; Norris et al., 2020; Byrne et al., 2021). However, these platforms generally remain material-based surrogates lacking biological functionality. Conversely, cell- and tissue-based vascular surrogates, including vessel-on-chip platforms and hybrid biofabricated constructs, incorporate living endothelium and multilayered cellular organization, enabling investigation of biocompatibility, thrombogenicity, and cell interaction under flow (Portone et al., 2024; Alvarez Castro et al., 2022; Radley et al., 2020). Despite their biological sophistication, these systems often rely on complex fabrication workflows, limited scalability, and reduced compatibility with standard endovascular procedures, leaving a methodological gap between procedurally robust and biologically relevant vascular models.

In this context, bioprinted vascular models are increasingly recognized as a promising intermediate solution, offering human-relevant, controllable, and customizable testbeds for benchmarking vascular devices before animal or clinical studies (Fazal et al., 2021; Kim et al., 2025). By leveraging imaging-derived geometries, bioprinting enables the reproduction of patient-specific anatomies, including bifurcations, stenoses, and aneurysm-like dilations, allowing device testing in realistic configurations (Miri et al., 2019; Ching et al., 2022; Son et al., 2025). Advances in hydrogel engineering have further enabled the fabrication of vessels with tunable compliance, burst pressure, and deformation behavior, supporting the evaluation of deployment forces, recoil, and wall interactions beyond what is achievable with rigid phantoms (Fazal et al., 2021; Zhang et al., 2021; Wang et al., 2022). In parallel, multilayer bioprinted vascular constructs integrating endothelial cells, smooth muscle cells, and fibroblasts have demonstrated increasing biological functionality, including endothelialization, thrombo-inflammatory responses, and direct vessel–device interaction under perfusion (Portone et al., 2024; Ching et al., 2022; Wang et al., 2022; Gold et al., 2021; Jiang et al., 2023; Sun et al., 2023; Perini, 2021).

To transition these models into reliable preclinical tools, critical biofabrication challenges must be addressed. Recent reviews consistently highlight that print reproducibility, shape fidelity, and mechanical consistency remain unresolved hurdles (Fazal et al., 2021; Jiang et al., 2023; Kim et al., 2023; Mir et al., 2023). Among the available biofabrication strategies, Freeform Reversible Embedding of Suspended Hydrogels (FRESH) bioprinting has emerged as an attractive approach owing to its ability to fabricate complex tubular geometries using low-viscosity hydrogels while preserving structural integrity during printing (Hinton et al., 2015). Recent advances in FRESH have also introduced quantitative, non-destructive volumetric imaging approaches, particularly optical coherence tomography (OCT), enabling *in situ* assessment of printing errors and dimensional fidelity (Tashman et al., 2023). More recently, quantitative metrology has been combined with high-fidelity FRESH fabrication of complex internally perfusable architectures (Shiwarski et al., 2025). These studies establish the feasibility of quantitative metrology within FRESH biofabrication; however, the systematic characterization of process-induced geometric changes across post-processing stages and, in particular, the mechanical reproducibility of independently fabricated batches remain comparatively underexplored. Although small deviations in lumen diameter or wall thickness can substantially alter perfusion hemodynamics and vessel-device interaction, integrating geometric process monitoring with explicit batch-to-batch mechanical assessment therefore represents an important step toward establishing reproducible vascular test platforms.

This technological gap is further compounded when scaling vascular models to different clinical dimensions. For instance, advanced biofabrication techniques, such as single-step coaxial microfluidic bioprinting, have shown outstanding success in fabricating small-caliber, biologically active vascular conduits (typically <2 mm in diameter) (Wang et al., 2022). However, these strategies face physical and geometric limitations when scaled up to medium- to large-caliber vessels (>5 mm). However, conventional coaxial extrusion strategies are primarily suited to continuous hollow conduits and can become technically challenging when reproducing spatially varying, non-uniform vascular geometries, such as localized stenotic narrowing. Furthermore, as highlighted in recent literature on embedded bioprinting, ensuring high shape fidelity and preventing structural collapse during extraction of such larger hydrogel constructs from their supporting matrices remain major challenges (Budharaju et al., 2024). Importantly, individual capabilities relevant to vascular testing have already been demonstrated in advanced biofabricated systems, including FRESH-based quantitative volumetric metrology (Tashman et al., 2023), high-fidelity FRESH fabrication integrated with perfusion (Shiwarski et al., 2025), and perfusion and intraluminal stent deployment in endothelialized bioprinted vascular constructs (Ahmad et al., 2024). Hybrid biofabricated vessels have also been proposed for intravascular medical device testing and dynamic perfusion (Portone et al., 2024). Thus, the remaining methodological need is not to demonstrate these capabilities individually, but to establish how they can be incorporated into a quantitatively characterized workflow centered on process control and manufacturing reproducibility.

In this context, the present study establishes a controlled and reproducible FRESH-bioprinted workflow for the fabrication and quantitative characterization of vessel-like constructs intended for device-oriented applications. The proposed approach prioritizes process robustness, geometric reproducibility, and mechanical consistency as enabling features towards standardized experimental testing, rather than aiming to replicate native arterial physiology. Bioink formulation, printing parameters, and post-processing steps were systematically optimized and quantitatively assessed using non-destructive micro-computed tomography (micro-CT). Micro-CT was applied at defined fabrication stages to quantify process-induced changes in construct geometry. At the same time, complementary mechanical tests were performed across independent fabrication batches to assess mechanical reproducibility directly. In addition, sterile bioprinting, preliminary cellularization, controlled perfusion, and intraluminal stent deployment were explored to demonstrate procedural compatibility. The biological component is integrated solely to demonstrate the compatibility of the controlled fabrication workflow with basic cell-housing and sterile-handling procedures. Rather than presenting these individual capabilities as stand-alone technological advances, the present study integrates them into a process-oriented framework centered on quantitative reproducibility in manufacturing. To the best of our knowledge, the combined assessment of geometric evolution across FRESH processing stages and mechanical reproducibility across independent fabrication batches, followed by demonstration of compatibility with perfusion and intraluminal device deployment, has not been systematically investigated within a single vascular biofabrication workflow. Overall, the proposed workflow provides a quantitatively characterized experimental foundation for future biological refinement and device-oriented validation.

## 2 Materials and methods

Figure 1 schematically summarizes the experimental workflow used in this study, linking fabrication steps to the corresponding geometric, mechanical, and biological characterization procedures.

**FIGURE 1:**
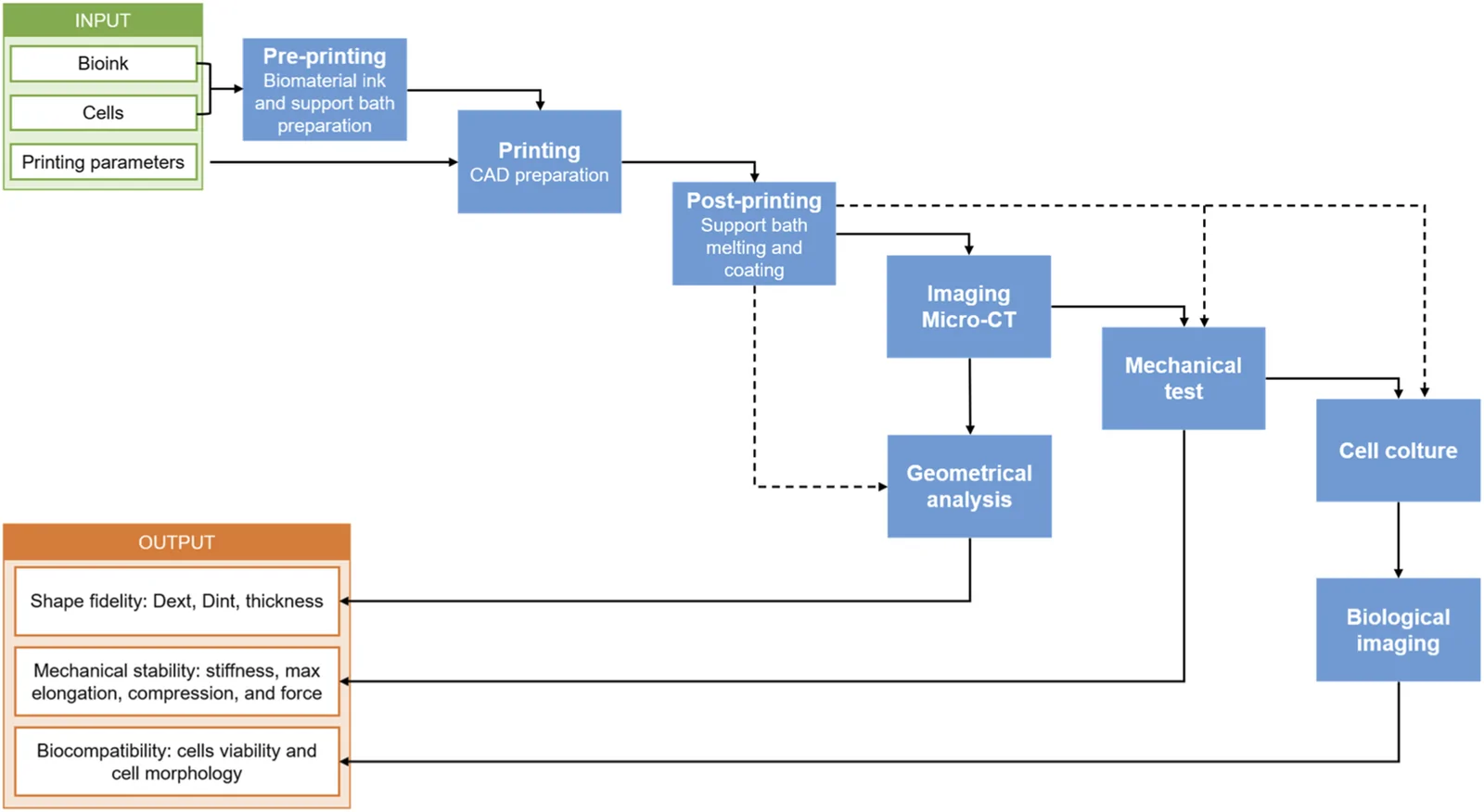
Schematic overview of the FRESH bioprinting and characterization workflow. The workflow begins with the definition of input parameters, including bioink formulation, cell incorporation, and printing settings, followed by pre-printing preparation of the biomaterial ink and support bath, CAD-based construct design and printing, and post-printing processing, including support bath melting and coating. Printed constructs are subsequently characterized through micro-CT imaging and geometrical analysis, mechanical testing, cell culture, and biological imaging. The workflow enables quantitative evaluation of shape fidelity (internal diameter, D_int_; external diameter, D_ext_; wall thickness), mechanical stability (stiffness, maximum elongation/compression, and maximum force), and biocompatibility (cell viability and morphology).

### 2.1 Biomaterial ink preparation

A low-viscosity hydrogel composed of sodium alginate (SA) and gelatin (GEL) was selected as a model formulation for systematic optimization of the fabrication workflow (Delgrosso et al., 2023; Fantini et al., 2019; Di Gravina et al., 2022; Scocozza et al., 2025). In this formulation, SA served as the primary structural component, while gelatin was included to improve printability and cell compatibility. GEL powder (Sigma-Aldrich, Italy) was dissolved in preheated (72 °C) sterile phosphate-buffered saline (PBS, EuroClone). SA powder (Sigma-Aldrich, Italy) was subsequently added and vortex-mixed until complete homogenization. The hydrogel was centrifuged at 2000 rpm for 5 min to remove air bubbles and pasteurized in a water bath at 72 °C for 1 h. The final mixture was transferred into syringes and stored at 4 °C until printing. Specifically, SA concentrations of 3% and 6% w/v were selected to span the low-to-medium viscosity range. Since our prospective developments aim to translate this workflow to highly diluted, low-viscosity ECM proteins (e.g., collagen), the 3% SA formulation was chosen to mimic the rheological challenges of these protein-based inks within the FRESH support bath. In comparison, the 6% SA served as a medium-viscosity benchmark for evaluating the trade-off among printability, structural resolution, and mechanical robustness. GEL concentration was held constant at 2% (w/v).

### 2.2 Biofabrication of a 3D vessel-like model

FRESH bioprinting workflow consists of three sequential phases—pre-printing, printing, and post-printing (Figure 1)—and was employed to fabricate tubular constructs from low-viscosity hydrogels. The workflow was systematically implemented to investigate the influence of fabrication parameters and post-processing steps on construct shape fidelity, geometric stability, and reproducibility.

#### 2.2.1 Pre-printing

The pre-printing phase consisted of preparing a gelatin-based support bath to provide temporary mechanical support during filament deposition and to stabilize low-viscosity bioinks during printing.

The support bath was prepared following the protocol proposed by Hinton et al. (2015), with minor modifications. Gelatin from bovine skin (Type B, Sigma-Aldrich, Italy) was dissolved in 150 mL of 1% (w/v) calcium chloride (CaCl_2_, Sigma-Aldrich, Italy) solution to obtain a final gelatin concentration of 4.5% (w/v) and stored at 4 °C for 24 h. Subsequently, 350 mL of cold CaCl_2_ solution was added, and the mixture was blended for 30 s. The slurry was transferred into 50 mL conical tubes and centrifuged at 4200 rpm for 3 min to allow particle sedimentation. The supernatant was removed, and the slurry was repeatedly resuspended and centrifuged until the supernatant appeared free of bubbles, indicating that excess soluble gelatin had been removed. The final support bath was transferred into printing containers and stored at 4 °C for up to 12 h before use.

The presence of CaCl_2_ in the support bath promoted ionic crosslinking of alginate during printing, contributing to the initial stabilization of deposited filaments.

#### 2.2.2 Printing

The printing phase involved extrusion-based deposition of the SA–GEL hydrogel within the support bath using a CELLINK INKREDIBLE + bioprinter (CELLINK AB, Sweden). The bioink was loaded into syringe cartridges equipped with Luer-lock connectors and extruded through needles with inner diameters of 0.4–0.6 mm (length: 1.5 cm).

Printing was performed at room temperature, while the printhead temperature was maintained at 30 °C to ensure stable extrusion behavior. Printing parameters, including extrusion pressure, nozzle diameter, and printing speed, were systematically optimized according to bioink composition and nozzle geometry to achieve continuous filament deposition, minimize structural deformation, and maximize construct shape fidelity.

#### 2.2.3 Post-printing

Following deposition, constructs remained embedded in the support bath and were incubated at 37 °C for 30 min (5% CO_2_, 95% humidity) to melt the gelatin support and release the printed structures. During printing, ionic crosslinking of alginate occurred within the CaCl_2_-containing support bath, providing initial stabilization of deposited filaments. However, to improve structural integrity and interlayer cohesion, an additional post-printing coating was introduced.

Each construct was sequentially immersed in a 1% (w/v) SA solution for 1 min, then in a 2% (w/v) CaCl_2_ solution for 2 min. This coating process promoted additional surface crosslinking and improved construct stability.

The post-processing workflow, including support bath melting and coating conditions, was systematically optimized to reduce geometric deformation and improve construct reproducibility and mechanical consistency.

### 2.3 Shape fidelity assessment by micro-CT

Micro-CT was used to quantitatively assess the geometry of FRESH-bioprinted constructs through non-destructive three-dimensional characterization of internal and external diameters, wall thickness, and overall morphology. Micro-CT acquisitions were performed at defined stages of the fabrication process, including immediately after printing (within the support bath) and after post-processing (following support bath removal and coating), to evaluate shape fidelity and geometric changes induced by processing steps.

#### 2.3.1 Sample preparation

Tubular constructs with a height of 10 mm and diameters of 5, 15, and 25 mm were designed and printed (Figure 2a). This diameter range enabled evaluation of fabrication fidelity across multiple geometrical scales relevant to vascular applications. Two nozzle diameters (0.4 and 0.6 mm) and two SA concentrations (3% and 6% w/v) were investigated, with three replicates per condition. Micro-CT scans were acquired at the fabrication stages described above to quantify geometrical variations associated with printing and post-processing. Each scan required approximately 15 min, and constructs of different diameters were scanned together, when possible, to ensure consistent acquisition conditions. Representative samples before and after support bath melting are shown in Figures 2b,c.

**FIGURE 2:**
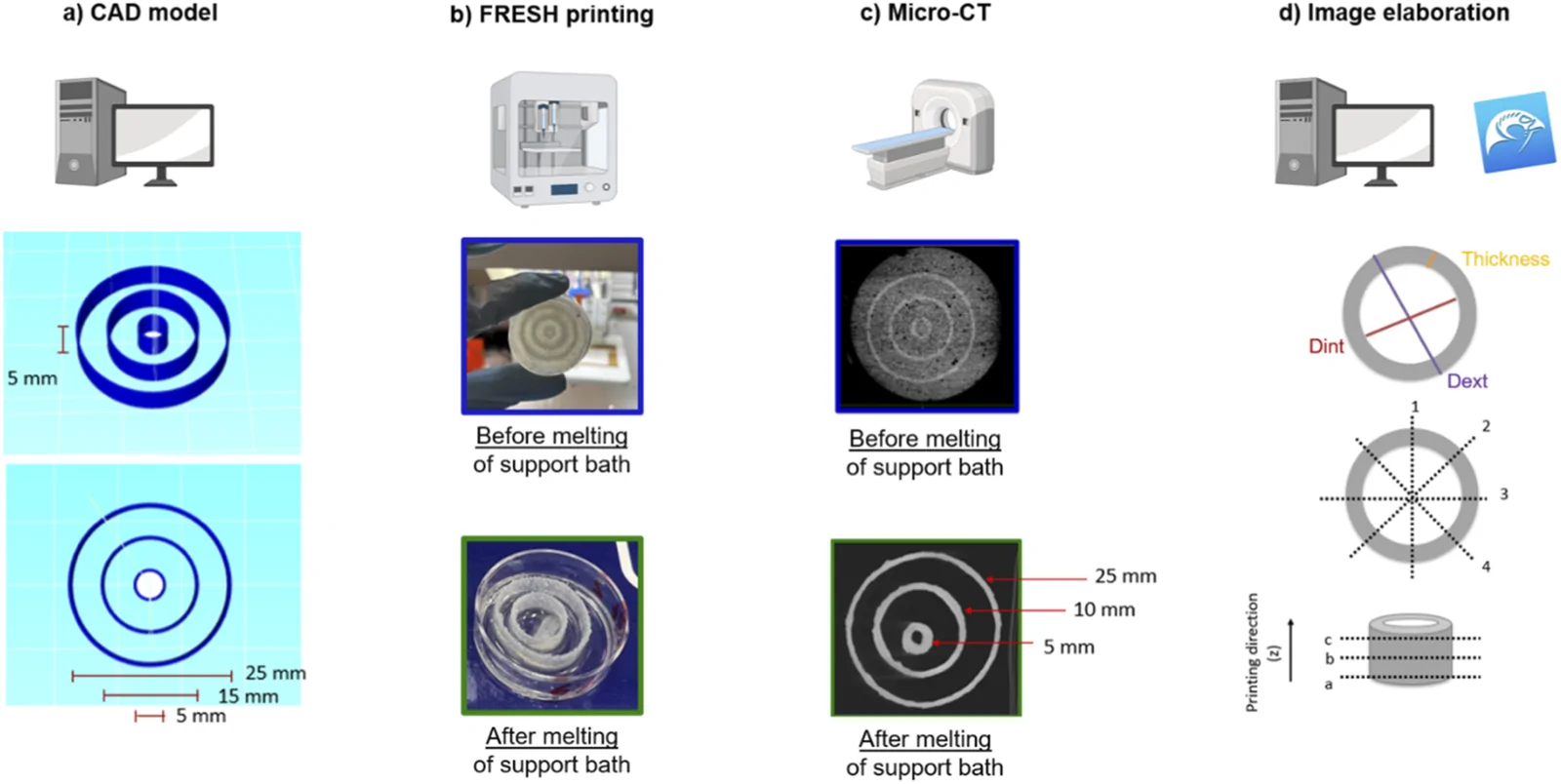
Workflow for quantitative shape-fidelity assessment of FRESH-bioprinted vascular constructs. **(a)** CAD design of tubular constructs (height: 10 mm) with three target diameters (5, 15, and 25 mm). **(b)** Representative FRESH-bioprinted constructs before and after bath melting. **(c)** Micro-CT imaging was performed before and after support-bath melting to evaluate geometric changes induced by post-processing. **(d)** Schematic representation of image-based geometrical analysis used to quantify internal diameter (D_int_), external diameter (D_ext_), and wall thickness from cross-sectional images. Measurements were performed at multiple angular positions within the x–y plane and across different sections along the longitudinal (z) axis to account for local geometrical variability.

#### 2.3.2 Acquisition and reconstruction

Micro-CT scans were performed using a SkyScan 1276 system (Bruker, Belgium). Acquisition parameters were selected to ensure adequate spatial resolution for quantitative geometrical measurements of the printed constructs.

Scanning settings included a voxel size of 40.67 µm, tube voltage of 45 kV, current of 200 μA, and a 0.25 mm aluminum filter. Images were acquired every 0.4° over a 180° rotation using 4 × 4-pixel binning. Each projection was acquired twice and averaged to reduce noise. For larger constructs, 2–3 overlapping scans were performed to ensure full sample coverage.

Raw projection data were reconstructed using NRecon software (Bruker) with Gaussian smoothing (level 2) and 40% beam-hardening correction. Ring-artifact and post-alignment corrections were individually adjusted for each dataset. Overlapping scans were automatically fused to generate continuous three-dimensional reconstructions, which were subsequently used for quantitative geometrical analysis.

#### 2.3.3 Image processing and geometrical analysis

Reconstructed 16-bit TIFF image stacks were imported into Horos (open-source medical image analysis software) for visualization and quantitative analysis. Image contrast and brightness were adjusted for visualization purposes only and did not affect quantitative measurements. Geometrical parameters, including internal (D_int_) and external (D_ext_) diameters, were measured from cross-sectional images. At the same time, wall thickness was calculated as: $\left(D_{ext}-D_{\mathrm{int}}\right)/2$ .

Measurements were performed on multiple slices along the length of the construct to account for local geometric variations. Mean values and standard deviations were calculated for each condition. Geometrical measurement definitions are illustrated in Figure 2d.

Three-dimensional reconstructions were generated using a region-of-interest (ROI)-based segmentation approach. ROIs were manually delineated on consecutive cross-sectional slices and interpolated when appropriate to obtain continuous contours. The resulting ROI stack was used to generate three-dimensional surface reconstructions for qualitative and quantitative evaluation of construct morphology.

### 2.4 Mechanical stability assessment

To evaluate the structural stability and reproducibility of FRESH-bioprinted constructs, confined uniaxial compression and ring tensile tests were performed as complementary mechanical assays. These tests were designed to characterize the mechanical response under axial and circumferential loading conditions and to quantify intra- and inter-batch variability. Mechanical analysis was conducted according to the methodology described by Casamao et al. (2021).

For confined compression testing, ring-shaped samples (diameter: 10 mm; height: 10 mm) were loaded using a custom fixture designed to prevent lateral expansion and isolate the axial compressive response. Tests were performed at a compression rate of 1 mm/min with a preload of 0.02 N.

For ring tensile testing, ring-shaped samples (diameter: 15 mm; height: 5 mm) were subjected to uniaxial tensile loading using a dedicated ring-testing setup. Tests were performed at a displacement rate of 10 mm/min with a preload of 0.02 N.

Three independent fabrication batches were evaluated for both confined compression and ring tensile testing, with five samples tested per batch (n = 15 per mechanical assay).

Force–displacement data were analyzed using a custom MATLAB script (MATLAB, The MathWorks, Inc., 2025). Raw force and displacement signals were converted to absolute values and smoothed using a moving-average filter to reduce high-frequency noise. The onset of the loading region was identified as the first continuously increasing segment that exceeded a predefined slope threshold, and the corresponding force and displacement values were used to rescale each curve to a common origin.

Structural stiffness was calculated as the slope of a least-squares linear regression fitted to the force–displacement data corresponding to 15%–30% engineering strain. Compression and elongation were expressed as percentages of the initial sample length or initial platen separation, depending on the test configuration. Maximum force and the corresponding maximum compression or elongation were extracted from the rescaled loading curve.

Engineering stress was calculated by normalizing force to the initial load-bearing cross-sectional area. For confined compression tests on hollow cylindrical specimens, the initial cross-sectional area was calculated as:

$$A_{0}=\frac{\pi }{4}\left(D_{ext}^{2}-D_{\mathrm{int}}^{2}\right),$$

where D_ext_ and D_int_ are the external and internal diameters, respectively. For ring tensile tests, the load-bearing cross-sectional area was calculated as:

$$A_{0}=2Wt,$$

where W is the axial width of the ring and t is the wall thickness; the factor of two accounts for the two opposite ring walls simultaneously carrying the tensile load.

Engineering strain was calculated as the displacement divided by the initial sample length or platen separation, while engineering stress was calculated as force divided by A_0_. Young’s modulus for the ring tensile test and the compressive modulus for the confined compression test were determined as the slope of a least-squares linear regression fitted to the engineering stress–strain curve between 15% and 30% strain. For each sample, stiffness, modulus, maximum force, and maximum compression or elongation were extracted. Results were averaged across repetitions within each batch and reported as mean ± standard deviation. Boxplots were generated to compare stiffness, maximum force, and maximum compression or elongation across batches. Detailed geometric parameters, including specimen dimensions, platen separation, wall thickness, diameters, and calculated cross-sectional areas, are reported in Supplementary Tables S1, S2 for the confined compression and ring tensile tests, respectively.

### 2.5 Cell-integrated vascular construct fabrication

The biological component of the workflow was designed to assess the compatibility of the FRESH process with basic cell culture procedures, including sterile fabrication, fibroblast encapsulation, and preliminary endothelial cell adhesion, rather than establishing a fully mature vascular tissue model.

#### 2.5.1 Sterile FRESH-bioprinted constructs

All printing steps were performed under sterile conditions using the FRESH technique. Gelatin powder and printing containers for support bath preparation were UV-irradiated (365 nm, 30 min), while CaCl_2_ solutions were sterile-filtered through 0.2 µm filters. Printing equipment, including needles and cartridges, was autoclaved before use. The bioprinter was placed under a sterile laminar flow hood for at least 30 min before printing.

Sterility of the workflow was assessed by bioprinting ring-shaped samples (inner diameter: 9 mm; height: 10 mm; n = 4) and incubating them in complete culture medium for 21 days. Samples were visually inspected throughout the incubation period for evidence of microbial contamination. Non-sterile printed constructs (n = 4) were used as controls.

#### 2.5.2 Fibroblast culture, bioink preparation, and viability assessment

Human fibroblasts (AG01522, Coriell Institute, Camden, NJ, US) were cultured in Dulbecco’s Modified Eagle Medium (DMEM, Lonza) supplemented with 15% fetal bovine serum (FBS, EuroClone) and 1% gentamicin at 37 °C in a humidified atmosphere containing 5% CO_2_. At approximately 80% confluence, cells were detached using Trypsin/EDTA, counted, and centrifuged at 1200 rpm for 10 min.

Fibroblasts were resuspended in culture medium and mixed with the SA–GEL hydrogel at a hydrogel-to-cell suspension ratio of 10:1, yielding a final cell density of 10 × 10^6^ cells/mL. For biological experiments, the optimized SA–GEL formulation identified during workflow optimization (6% w/v SA, 2% w/v GEL) was used. The resulting bioink was homogenized using a dual-syringe mixing system, loaded into printing cartridges, and briefly centrifuged to remove air bubbles.

Ring-shaped samples (inner diameter: 10 mm; height: 5 mm; n = 9) were bioprinted and cultured at 37 °C and 5% CO_2_. Cell viability was assessed after 24 h, 7 days, and 14 days (n = 3 per time point) using a Live/Dead viability assay (Invitrogen). Samples were incubated in staining solution for 20 min at 37 °C, protected from light, rinsed with PBS, and imaged using a Leica Thunder Live Cell Imager DMi8 fluorescence microscope.

Three random fields per construct were analyzed using Fiji/ImageJ software. Cell viability was calculated as:

$$\%\text{ Live cells}=\frac{\text{Live}\;\text{cells}}{\text{Total}\;\text{cells}}\times 100$$

Results are reported as mean ± standard deviation (SD) for n = 3 samples.

To evaluate fibroblast viability in a more biologically permissive matrix, the assay was repeated using a type I collagen bioink (LifeInk, Advanced Biomatrix, US). The collagen formulation was prepared according to the manufacturer’s instructions and used at a final concentration of approximately 18 mg/mL. For collagen-based printing, the FRESH support bath was prepared using Hank’s Balanced Salt Solution (HBSS).

#### 2.5.3 Evaluation of HUVEC adhesion on fibroblast-laden constructs

Following fibroblast encapsulation and viability assessment, endothelial cell adhesion was evaluated to assess the constructs’ compatibility with endothelial cell seeding. Human umbilical vein endothelial cells (HUVECs, Innoprot, Spain) were seeded onto fibroblast-laden ring-shaped constructs.

HUVECs were cultured according to the manufacturer’s protocol in Endothelial Cell Medium (Innoprot, Spain), consisting of basal medium supplemented with endothelial growth supplements, FBS, and antibiotics. Cells were maintained at 37 °C in a humidified atmosphere containing 5% CO_2_ and used at low passage numbers.

To enable cell tracking, fibroblasts and HUVECs were fluorescently labeled using CellTracker™ dyes (Invitrogen) according to the manufacturer’s instructions. Fibroblasts and HUVECs were labeled with green and red fluorescent dyes, respectively. Cells were incubated in serum-free medium containing the appropriate dye for 30 min at 37 °C, then detached with Trypsin/EDTA. Labeled fibroblasts were encapsulated within the SA–GEL hydrogel and bioprinted as described in Section 2.5.2. Four constructs were bioprinted and analyzed up to 21 days of culture.

HUVECs were resuspended at a density of 600,000 cells per construct in a total volume of 40 µL. The cell suspension was sequentially applied to the luminal surface, with the construct rotated by 90° after each 20 min incubation period to promote homogeneous cell distribution along the lumen. This procedure was repeated four times.

### 2.6 Perfusion capability and structural integrity under flow

To evaluate procedural compatibility for perfusion and intraluminal device deployment, a custom coupling module was developed to connect the bioprinted vessel-like constructs to a controlled-flow system while minimizing handling and preserving sterility.

The coupling module consisted of a chamber with internal housing designed to accommodate the construct, together with inlet and outlet connectors. Tubular samples (inner diameter: 10 mm; height: 5 mm; n = 9) were bioprinted, positioned within the chamber, and connected to the perfusion lines. To improve sealing between the construct and the connectors, two lateral walls divided the chamber into three compartments, and a 40% (w/v) Pluronic solution was applied to the outer compartments. The coupling module was designed using Autodesk Inventor. The main structural components were fabricated using fused deposition modeling (FDM), while connectors were produced via stereolithography (SLA) to improve geometric accuracy. Preliminary static perfusion tests were performed to assess system sealing and leakage.

To evaluate construct stability under flow, a perfusion circuit was assembled by connecting the coupling module to silicone tubing, with pressure sensors positioned upstream and downstream of the construct. Perfusion was performed using a syringe pump operating at a constant flow rate of 30 mL/min, with deionized water at room temperature (∼22 °C) used as the working fluid. The system was maintained under steady-flow conditions for 30 min. These conditions were selected to assess leakage, structural integrity, and procedural compatibility under controlled flow and were not intended to reproduce physiological hemodynamic or arterial wall shear stress conditions.

### 2.7 Vessel–device interaction: stent-like ring deployment

Intraluminal device compatibility was evaluated by deploying a radiopaque ring-shaped polycaprolactone (PCL) stent within the lumen of the bioprinted construct. This experiment was designed to investigate the mechanical interaction between the stent-like ring and the compliant hydrogel vessel wall. To qualitatively evaluate the potential of the fabricated vascular construct for endovascular applications, a proof-of-concept deployment test was performed using a 3D-printed self-expanding PCL ring. Specifically, instead of using a balloon catheter, the ring was compressed, loaded into a syringe (acting as a delivery sheath), and deployed into the vessel lumen. Following deployment, micro-CT was performed to evaluate ring positioning and integration within the construct lumen.

#### 2.7.1 Micro-CT image processing and segmentation

Micro-CT datasets were processed to generate high-resolution three-dimensional reconstructions of the bioprinted vascular constructs following ring deployment. Volumetric datasets were segmented using the open-source software ITK-SNAP to isolate both the hydrogel-based vessel and the PCL ring structure. Semi-automatic segmentation was performed using intensity thresholding combined with manual refinement to ensure accurate delineation of both components.

Segmented structures were exported as triangulated surface meshes (STL format) and preprocessed to remove artifacts and ensure spatial consistency before quantitative analysis.

#### 2.7.2 Quantitative geometrical analysis

A custom MATLAB pipeline was developed to evaluate cross-sectional geometrical features along the longitudinal axis of the construct. The principal axis of the vessel was identified using principal component analysis (PCA), and planar cross-sections were extracted orthogonal to the construct length.

To minimize boundary effects associated with partial intersections near the ring edges, ten equally spaced cross-sections were selected within the central ring region, excluding a 1 mm margin at both proximal and distal ends. For each slice, maximum (D_max_) and minimum (D_min_) diameters were calculated, and eccentricity was defined as:

$$E=\frac{D_{\max}-D_{\min}}{D_{\max}}$$

Changes in vessel diameter and eccentricity within the ring region were quantified relative to reference regions outside the ring to evaluate local expansion and ovalization induced by the implanted structure.

### 2.8 Statistical analysis

For micro-CT analyses, comparisons across processing stages were performed separately for each fabrication condition. Because the same constructs were imaged before support-bath melting, after melting, and after coating, external diameter, internal diameter, and wall thickness were analyzed using one-way repeated-measures ANOVA, followed by corrected pairwise comparisons when appropriate. Mechanical parameters across independent fabrication batches were analyzed using one-way ANOVA followed by Tukey’s multiple-comparison test. Differences were considered statistically significant at p < 0.05.

## 3 Results

Results are presented following the progression of the proposed workflow, from fabrication optimization and quantitative characterization to evaluation of procedural compatibility. Printing parameters were first optimized to improve construct reproducibility, followed by geometrical and mechanical assessment. Finally, sterile fabrication, cellularization, perfusion, and intraluminal stent deployment were explored as preliminary demonstrations of platform compatibility for device-oriented vascular testing.

### 3.1 Optimization of the FRESH printing process parameters

A systematic screening of FRESH bioprinting parameters was performed to identify fabrication conditions that minimize filament collapse and geometric deformation while ensuring stable construct formation. The optimization strategy was structured into two sequential steps: (i) optimization of global process parameters, including support bath composition, printing conditions, and post-processing settings, and (ii) optimization of extrusion pressure as a function of nozzle diameter and SA–GEL composition. The optimized parameters are summarized in Tables 1 and 2.

**TABLE 1:** Fixed parameters for the FRESH-bioprinted process. RT: room temperature.

Category	Parameters	Ranges tested	Optimized value
Support bath preparation	CaCl_2_ concentration	0.33%–1% (w/v)	0.33%
Printing parameters	Printing speed	5–15 mm/s	10 mm/s
	Printing pressure	10–60 kPa	45 kPa
	Print-head temperature	RT – 30° C	30° C
Construct release	Time of bath melting	30–60 min	45 min
Coating	Time of coating and crosslinking	30 s–1 min coating 1–2 min crosslinking	1 min coating +2 min crosslinking

**TABLE 2:** Optimized extrusion pressures for sodium alginate (SA) concentrations and needle size.

SA concentration	Needle internal diameter	Pressure ranges tested	Optimized value
3% (w/v)	0.41 mm	10–25 kPa	15 kPa
	0.61 mm	5–20 kPa	10 kPa
6% (w/v)	0.41 mm	30–60 kPa	50 kPa
	0.61 mm	20–40 kPa	30 kPa

In the first optimization step, support bath composition, printing speed, printhead temperature, support bath melting time, and coating conditions were systematically adjusted to ensure stable filament deposition, adequate interlayer support, reproducible construct release, and preservation of structural integrity following post-processing.

The second optimization step focused on extrusion pressure, which emerged as one of the most influential parameters governing filament continuity and shape fidelity. For each bioink–nozzle combination, a pressure range was systematically evaluated to identify the minimum pressure required to ensure continuous filament extrusion without over-deposition or structural deformation. The optimal pressure increased with both alginate concentration and nozzle diameter, reflecting greater flow resistance due to increased hydrogel viscosity and geometric constraints during extrusion.

Under non-optimized conditions, constructs frequently exhibited filament collapse, wall discontinuities, and loss of tubular geometry. In contrast, optimized parameters enabled smooth filament deposition and continuous wall formation while preserving the intended architecture (Figure 3a). For example, constructs fabricated using 6% (w/v) SA with a 0.41 mm nozzle at 30 kPa exhibited partial filament collapse, whereas increasing the extrusion pressure to 50 kPa resulted in stable tubular formation and improved geometric definition. Similar trends were observed across all tested bioink–nozzle combinations.

**FIGURE 3:**
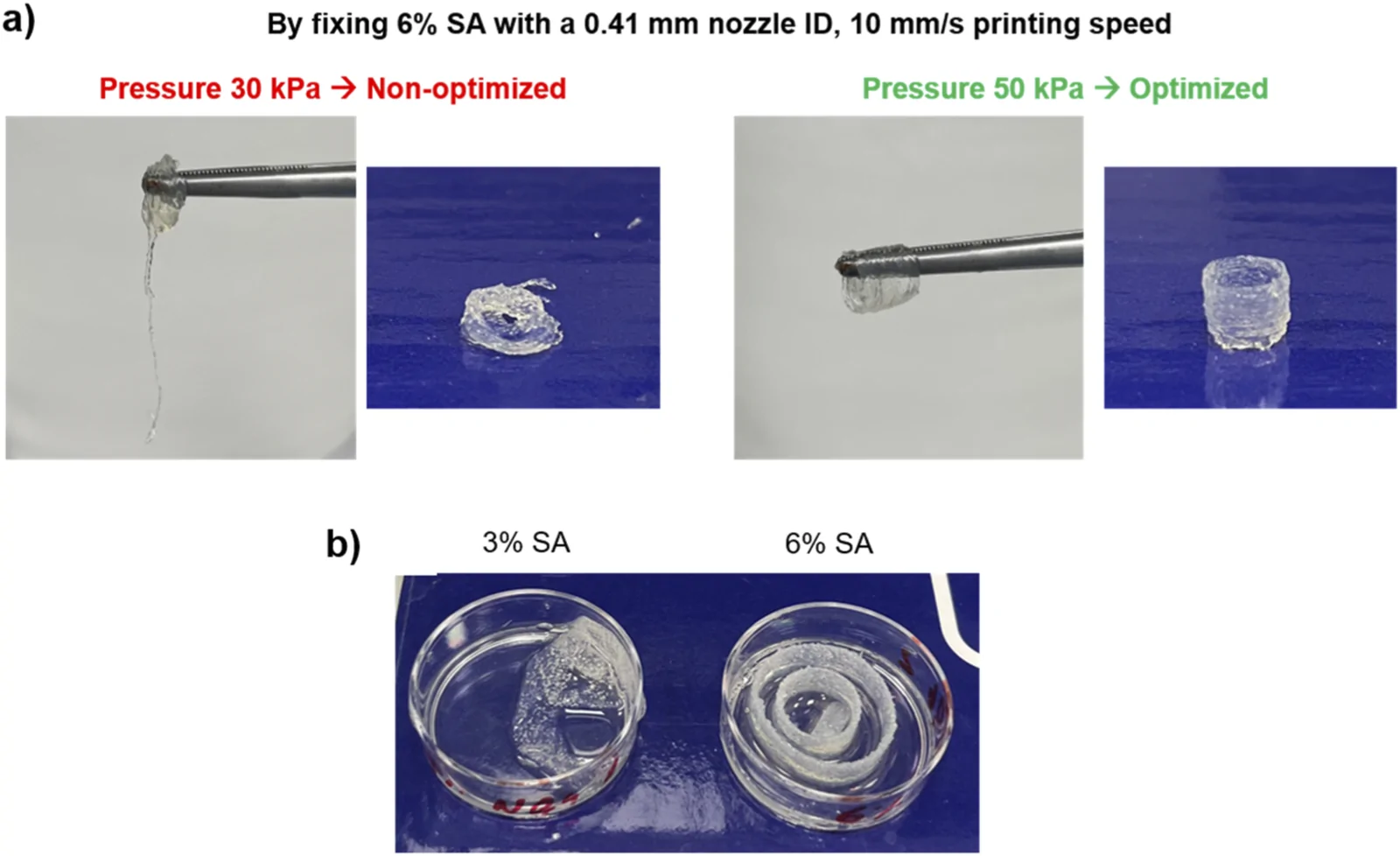
Optimization of extrusion pressure and influence of alginate concentration on construct fidelity. **(a)** Representative effect of extrusion pressure on the printability of constructs fabricated using 6% (w/v) sodium alginate (SA) and a 0.41 mm nozzle. Left: non-optimized extrusion pressure (30 kPa) led to filament collapse and poor geometric definition. Right: optimized pressure (50 kPa) enabled stable filament deposition and formation of continuous tubular structures. **(b)** Representative constructs fabricated using 3% (left) and 6% (right) SA following support bath melting. The 3% SA formulation exhibited reduced structural stability and limited handling robustness, whereas the 6% SA formulation maintained structural integrity and preserved tubular geometry.

Marked differences were also observed between bioink formulations. Constructs fabricated with 3% (w/v) SA showed limited structural stability, particularly for larger geometries (e.g., 25 mm diameter), where deformation and reduced handling robustness were frequently observed (Figure 3b). In contrast, the 6% (w/v) SA formulation enabled reproducible fabrication across all tested geometries and was therefore selected for subsequent mechanical, geometrical, and biological characterization.

### 3.2 Shape fidelity assessment by micro-CT

Quantitative micro-CT analysis was performed to assess shape fidelity and geometrical reproducibility of the FRESH-bioprinted constructs and to evaluate the influence of bioink composition, nozzle geometry, and post-processing on final construct dimensions. Micro-CT was employed here to provide high-resolution three-dimensional characterization of vessel-like constructs across fabrication stages and geometrical scales.

Constructs printed using 6% (w/v) SA showed close agreement between designed and measured internal (D_int_) and external (D_ext_) diameters, with relatively low standard deviations across all tested geometries (Figures 4a,b). Wall thickness values were consistently slightly lower than target dimensions and were largely independent of nozzle diameter.

**FIGURE 4:**
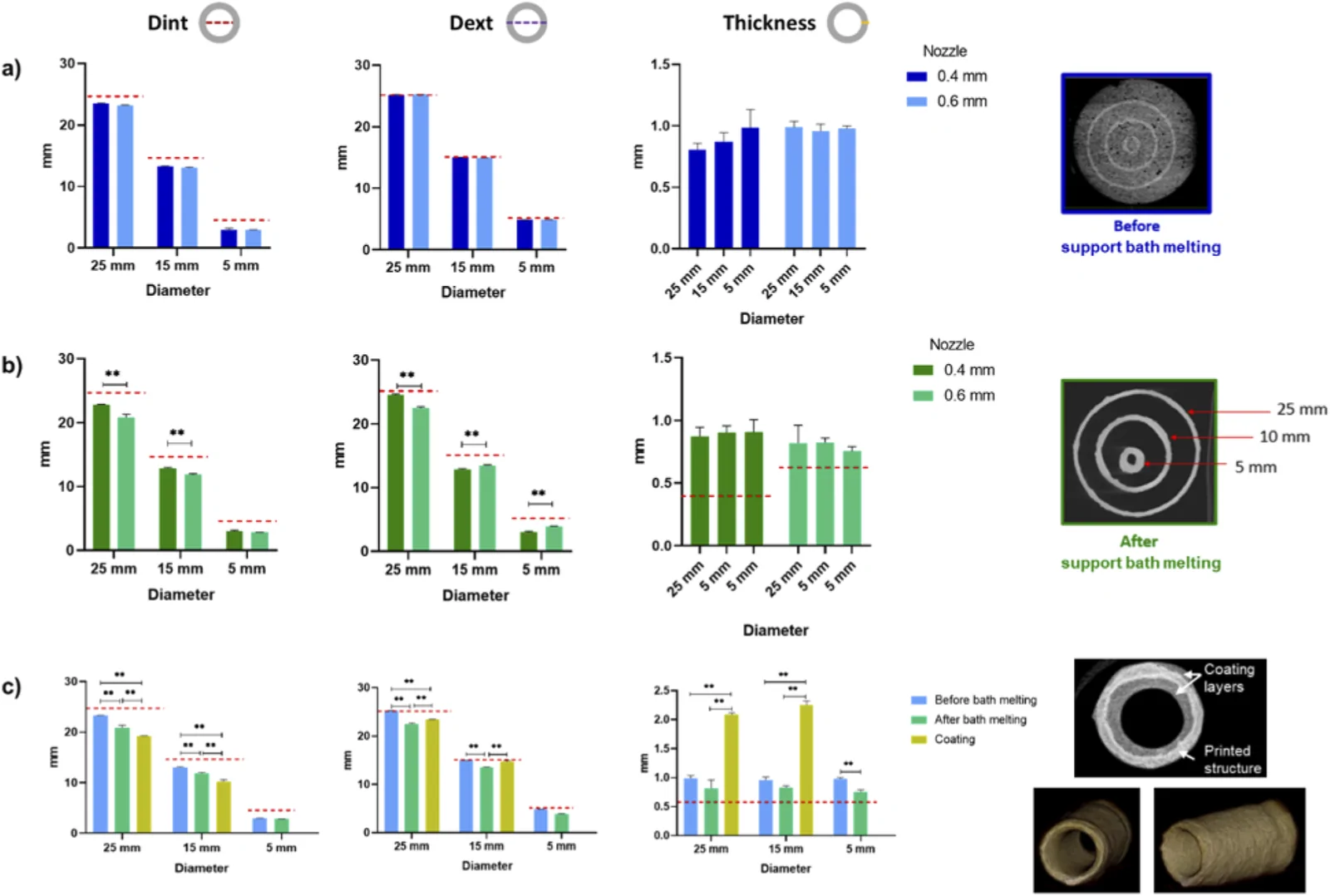
Quantitative micro-CT assessment of FRESH-bioprinted construct shape fidelity before and after post-processing. Geometrical measurements of internal diameter (D_int_), external diameter (D_ext_), and wall thickness for constructs fabricated using 6% (w/v) SA. **(a)** Measurements obtained before support bath melting, comparing constructs printed with 0.4 mm and 0.6 mm needle nozzles. **(b)** Measurements obtained after support bath melting, showing slight shrinkage in D_int_ and D_ext_, particularly for 15- and 25-mm constructs. **(c)** Effect of the post-printing coating procedure on construct geometry for samples printed with a 0.6 mm nozzle, demonstrating lumen reduction and increased wall thickness. For 5-mm constructs, coating partially obstructed the lumen, preventing reliable geometrical measurements. Representative micro-CT images corresponding to each processing stage are shown on the right. Red dashed lines indicate target geometrical values. Error bars represent standard deviation. **p < 0.01.

In contrast, constructs fabricated with 3% (w/v) SA exhibited limited structural stability and poor robustness to handling, particularly for larger geometries (e.g., 25 mm diameter). Moreover, micro-CT segmentation was hindered by overlapping grayscale histograms between the construct and the surrounding environment (Supplementary Figure S1), preventing reliable geometrical quantification. Based on these observations, subsequent micro-CT analyses were restricted to constructs fabricated with 6% SA.

Post-processing steps significantly influenced construct geometry. Support bath melting induced a slight but statistically significant reduction (p-value<0.01) in both D_ext_ and D_int_ for constructs with diameters of 15 and 25 mm, whereas 5-mm constructs remained largely unaffected (Figure 4c). Subsequent coating further reduced lumen diameter while increasing wall thickness. In certain conditions, particularly with 5-mm constructs and samples printed with 0.4-mm nozzles, the coating partially obstructed the lumen, preventing reliable post-coating measurements.

### 3.3 Mechanical stability assessment

Mechanical testing was performed to assess structural stability, batch-to-batch reproducibility, and sensitivity of the constructs to fabrication and post-processing steps. The mechanical response of the FRESH-bioprinted constructs was evaluated through complementary confined compression and uniaxial ring tensile tests (Figure 5), enabling characterization under both axial and circumferential loading conditions.

**FIGURE 5:**
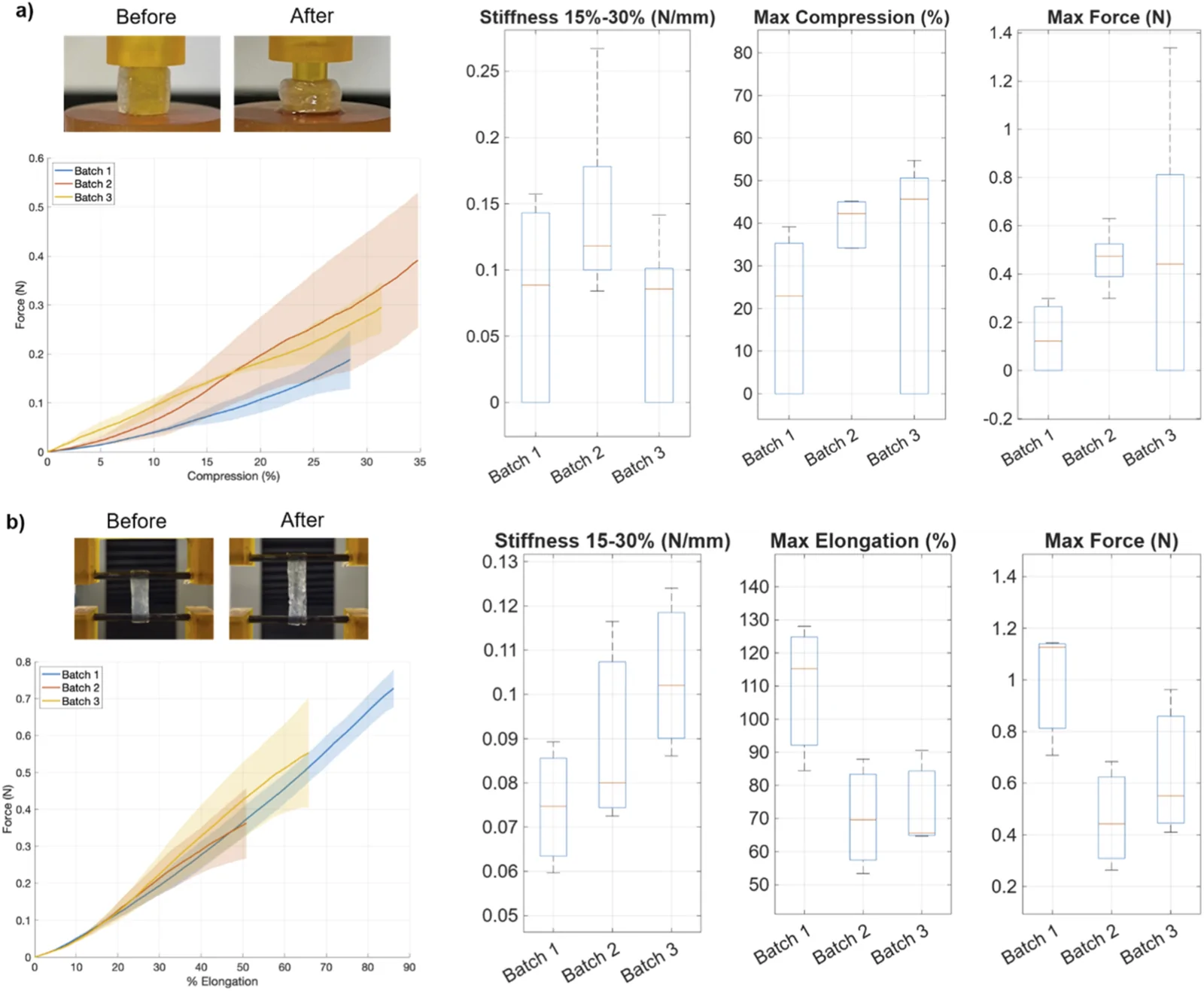
Mechanical characterization of FRESH-bioprinted ring-shaped constructs under confined compression and uniaxial tensile loading. Mechanical data are presented as force-displacement curves to assess batch-to-batch reproducibility in the overall testing platform. **(a)** Confined compression testing. Representative images of the testing setup are shown above the corresponding mean force–compression (mean ± standard deviation; n = 5 per batch). Boxplots summarize stiffness, calculated by linear regression between 15% and 30% compressive strain, maximum compression, and maximum force. The results indicate reproducible compressive behavior across batches, with moderate variability primarily associated with post-processing, particularly the coating step. **(b)** Ring tensile testing. Representative images of the tensile setup are shown above the corresponding mean force–elongation curves (mean ± standard deviation; n = 5 per batch). Boxplots summarize stiffness, calculated by linear regression between 15% and 30% compressive strain, maximum elongation, and maximum force, indicating reproducible circumferential mechanical behavior across fabrication batches. Detailed specimen dimensions and calculated load-bearing cross-sectional areas are reported in Supplementary Tables S1, S2 for confined compression and ring tensile testing, respectively.

Confined compression testing yielded a structural stiffness of 0.095 ± 0.044 N/mm, calculated from the linear region between 15% and 30% engineering strain, and a compressive modulus of 0.034 ± 0.01 MPa. Maximum compression between 19% and 40%, and maximum force between 0.13 and 0.48 N (Figure 5a). Mechanical variability among independent fabrication batches was generally limited, although slightly higher within individual batches. Despite these differences, the overall mechanical response remained consistent across batches.

Uniaxial ring tensile testing confirmed a reproducible circumferential mechanical response across independent batches. Force–elongation curves consistently exhibited an initial linear-elastic region, followed by progressive nonlinear deformation (Figure 5b). The structural stiffness, calculated between 15% and 30% engineering strain, was 0.0894 ± 0.0147 N/mm, while the corresponding Young’s modulus was 0.0358 ± 0.0031 MPa. Maximum elongation varied between 60% and 130%, and maximum forces ranged from 0.4 to 1.2 N. Variability remained limited at low deformation levels but increased moderately at higher elongations.

Preliminary long-term assessments conducted under culture conditions (37 °C, 5% CO_2_) up to 20 days showed a slight, non-significant reduction in mechanical properties (Supplementary Figure S2).

### 3.4 Cell-integrated vascular construct fabrication

The effectiveness of the sterile FRESH bioprinting workflow was first assessed by monitoring printed constructs for microbial contamination during long-term incubation. No visible signs of contamination were observed in sterile-printed samples for up to 20 days in complete culture medium, whereas non-sterile controls exhibited contamination.

Fibroblast viability and morphology were subsequently evaluated in constructs bioprinted using both SA–GEL and collagen-based bioinks at 24 h, 7 days, and 14 days (Figure 6a). Constructs fabricated with SA–GEL maintained high cell viability (>98%) throughout the culture period. However, fibroblasts consistently exhibited a rounded morphology with limited spreading. In contrast, collagen-based constructs supported high cell viability (>85%) and a markedly elongated, spread fibroblast morphology.

**FIGURE 6:**
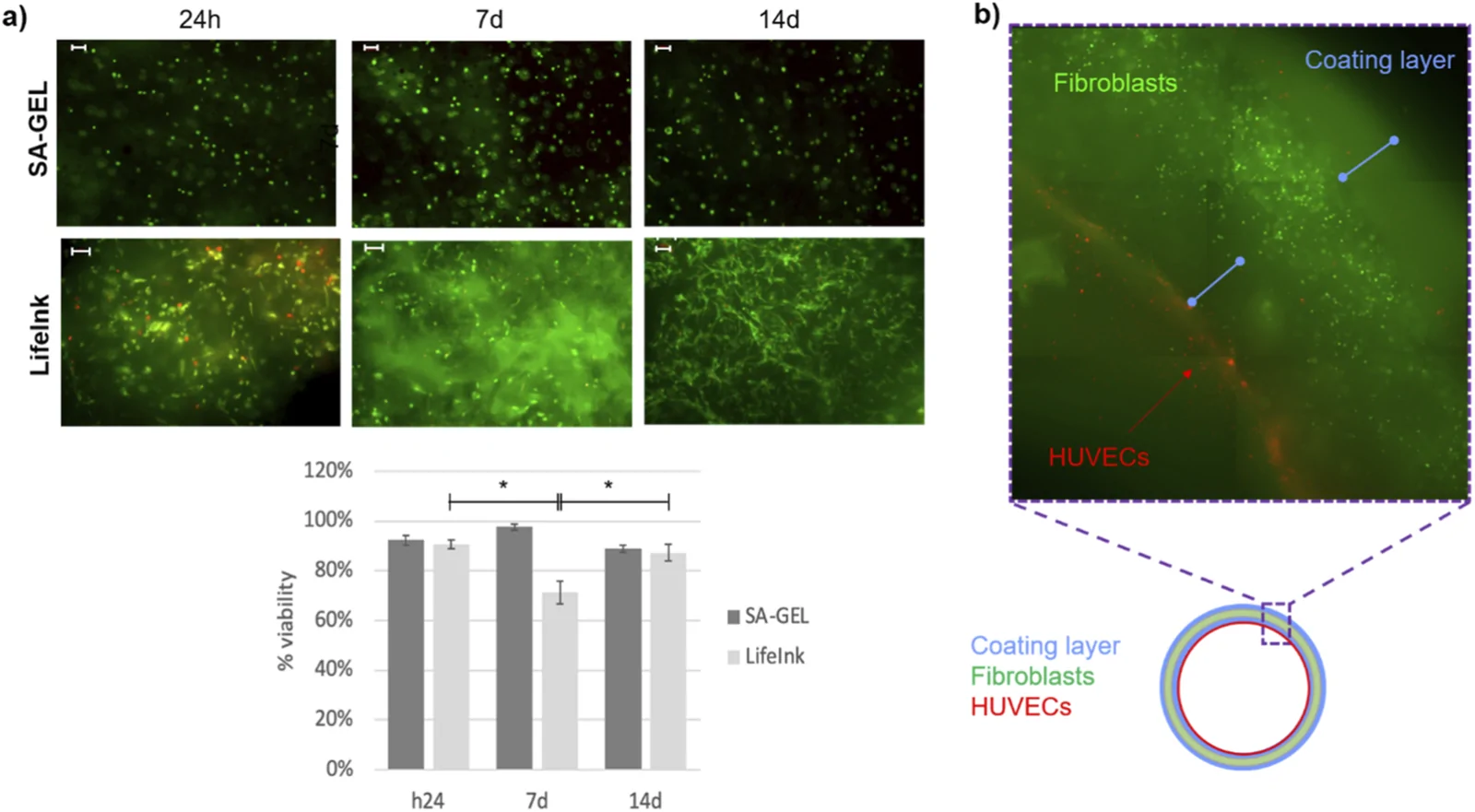
Biological characterization of FRESH-bioprinted vascular constructs. **(a)** Cell viability of fibroblast-laden constructs fabricated using SA–GEL and collagen-based (LifeInk) bioinks at 24 h, 7 days, and 14 days, assessed by Live/Dead staining (live cells: green; dead cells: red). Representative fluorescence images are shown above the corresponding quantitative analysis. SA–GEL constructs maintained high viability (>98%) up to 14 days throughout the culture period, whereas collagen-based constructs showed viability >85% with a transient decrease at day 7. **(b)** Representative fluorescence image of endothelialization constructs showing fibroblasts (green) embedded within the hydrogel matrix and HUVECs (red) localized along the luminal surface. Blue annotations highlight the coating layer. The schematic below illustrates the construct’s cross-sectional organization and indicates the imaged region. Scale bar: 70 µm *p < 0.05.

Following confirmation of fibroblast viability, endothelial cell adhesion was assessed by seeding HUVECs onto fibroblast-laden constructs. Fluorescence imaging revealed viable HUVEC adhesion along the luminal surface, with partial but distinct endothelial coverage of the inner wall (Figure 6b). A fully confluent endothelial monolayer was not achieved, but the observed cell attachment demonstrated coverage of the construct lumen.

### 3.5 Perfusion capability and structural integrity under flow

The bioprinted vascular construct was successfully integrated into the custom coupling module, with no evidence of leakage at the connection interfaces (Supplementary Figure S3). The perfusion setup enabled stable integration of the construct into the flow circuit while minimizing handling and preserving structural integrity.

Under baseline perfusion conditions (30 mL/min), corresponding to an intraluminal pressure of approximately 6–7 mmHg, the vessel-like construct maintained structural integrity and supported continuous flow without visible leakage.

Progressive pressurization, induced by partial outlet occlusion, increased intraluminal pressure up to approximately 24 mmHg. Above this threshold, fluid leakage occurred, accompanied by a transient pressure peak followed by a subsequent pressure decrease. Re-establishing baseline perfusion restored stable flow, suggesting that leakage was primarily related to hydrogel permeability rather than construct rupture or coupling module failure.

### 3.6 Vessel–device interaction: stent-like ring deployment

The radiopaque polycaprolactone (PCL) ring was successfully deployed within the lumen of the bioprinted construct without evidence of rupture, collapse, or visible structural damage to the hydrogel wall (Figure 7a). Upon release, the self-expanding structure successfully recovered its shape, exerting radial force on the vessel wall. The vascular construct accommodated the resulting local deformation without visible structural damage or coating delamination. This preliminary test demonstrates the compatibility of the bioprinted construct with deployment of the investigated self-expanding PCL stent-like ring under the tested conditions.

**FIGURE 7:**
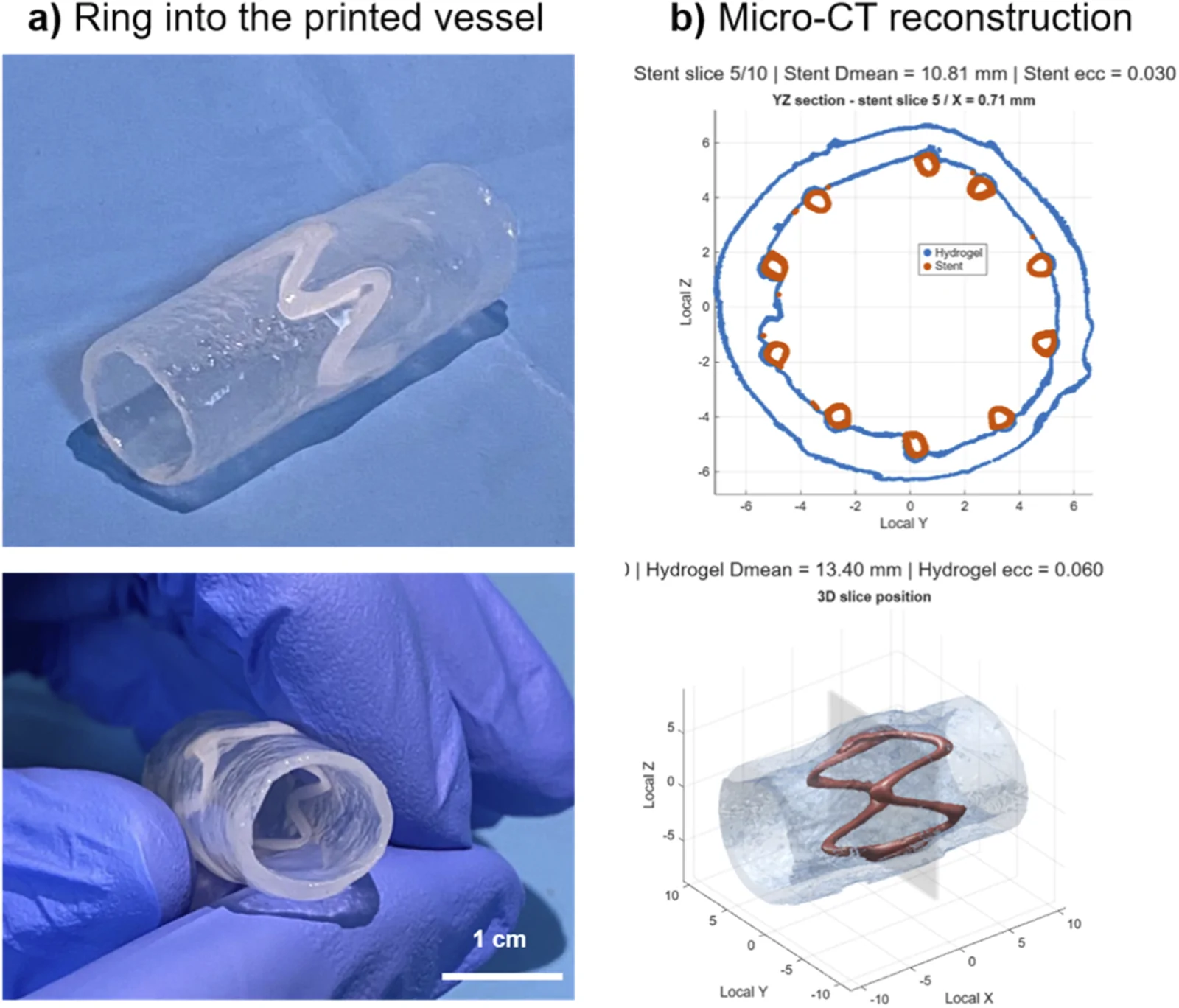
Vessel–device interaction following deployment of a PCL stent-like ring within the bioprinted vascular construct. **(a)** Representative images of the vascular construct after intraluminal deployment of the polycaprolactone (PCL) stent-like ring, showing stable positioning within the lumen and preservation of construct integrity. **(b)** Quantitative micro-CT analysis of ring deployment. A representative cross-sectional analysis illustrates geometric measurements of the hydrogel lumen and the implanted ring, including diameters and eccentricities from orthogonal slices. The three-dimensional reconstruction below shows the spatial relationship between the segmented vascular construct and the implanted PCL ring, enabling quantitative assessment of local construct expansion and ring-induced deformation.

Micro-CT enabled quantitative assessment of ring positioning and local geometrical changes induced by implantation. Figure 7b illustrates a representative cross-sectional slice, along with the geometric parameters used to evaluate local expansion and ovalization.

Quantitative analysis revealed a clear local mechanical effect of the implanted ring on the hydrogel construct. The unstented reference region exhibited a mean diameter of 12.18 mm and a low eccentricity index (0.065). Within the ring region, the hydrogel exhibited a consistent increase in diameter, reaching a mean value of 13.15 mm and a maximum of 13.45 mm, corresponding to an average radial expansion of approximately 7.9%, with local increases exceeding 10% relative to the reference configuration. Despite this expansion, lumen circularity was largely preserved, as indicated by a comparable mean eccentricity in the stented region (0.063). Localized increases in eccentricity (up to 0.12) suggest mild regional ovalization.

The PCL ring exhibited a mean deployed diameter of 10.61 mm, corresponding to approximately 88.4% of its nominal 12-mm diameter, together with a mean eccentricity of 0.107 (peak = 0.59). This constrained deployed configuration was associated with the localized lumen expansion described above while largely preserving lumen circularity.

## 4 Discussions

The main contribution of this work is the establishment of a quantitatively characterized, process-oriented workflow for reproducible manufacturing and characterization of FRESH-bioprinted vascular models suitable for device-oriented investigations. By systematically addressing the technical gap between rigid, non-biological vascular phantoms and highly complex, variable tissue-engineered systems, we demonstrated that process optimization combined with advanced 3D imaging can deliver structurally sound and geometrically accurate vascular models. Our findings show that the balance between bioink formulation and printing parameters is paramount for mitigating filament collapse and ensuring stable construct fabrication across multiple geometric scales. The structural instability observed in 3% (w/v) SA constructs is likely attributable to the lower viscosity and reduced mechanical support associated with the lower alginate concentration, which compromised filament stability during both deposition and post-processing. Conversely, the optimized 6% (w/v) SA formulation enabled reproducible fabrication, establishing a robust basis for subsequent quantitative characterization and device-oriented testing. Regarding wall thickness, the smaller-diameter constructs (5 mm) exhibited a higher relative dispersion due to the combined effects of FRESH bioprinting resolution limits and the manual coating step on highly curved surfaces. Nevertheless, the final achieved thickness (∼2 mm) closely matches the physiological scale of healthy native human arteries, which typically measure under 3 mm (Erbel and Eggebrecht, 2006). Furthermore, while the vascular constructs in this study were fabricated using a single-path extrusion followed by coating, the CAD/CAM-based approach could be extended to multiple concentric toolpaths to increase wall thickness or introduce spatially varying geometrical features, including localized stenotic profiles.

To understand the physical phenomena governing these printed structures, our micro-CT analysis revealed that the geometry of the constructs is strongly influenced by the interplay among bioink formulation, printing parameters, and post-processing conditions. Previous studies have established the value of non-destructive volumetric imaging for quantitative metrology in FRESH bioprinting, including *in situ* OCT-based assessment of printing errors and dimensional fidelity (Tashman et al., 2023) and, more recently, metrological characterization of high-resolution internally perfusable FRESH architectures (Shiwarski et al., 2025). Building on these advances, the present study uses micro-CT specifically as a process-monitoring tool to quantify geometrical evolution between defined fabrication and post-processing states. The discrepancies between targeted and measured wall thicknesses are likely attributable to a combination of extrusion pressure, viscoelastic filament relaxation after deposition, and partial radial compaction during ionic crosslinking within the CaCl_2_-containing support bath (Scocozza et al., 2025; Camasão and Mantovani, 2021). Furthermore, the shrinkage observed during support bath melting is likely associated with continued ionic crosslinking during incubation, which may induce progressive hydrogel compaction (Erbel and Eggebrecht, 2006; Achilli and Mantovani, 2010). Thus, the contribution of micro-CT in the present workflow does not reside in volumetric imaging *per se*, but in its integration with process-stage comparison to identify fabrication-induced dimensional changes and support reproducibility assessment.

From a mechanical standpoint, the observed variability appeared to be primarily associated with the manual coating procedure, which directly affects wall thickness and, consequently, construct stiffness and deformation behavior. Within the proposed workflow, this variability provides insight into the sensitivity of construct mechanics to post-processing conditions, highlighting opportunities for further process standardization. Compared with confined compression testing, ring tensile analysis demonstrated greater sensitivity to batch-to-batch differences. Importantly, whereas mechanical characterization of bioprinted vascular constructs has been widely reported, the present analysis was specifically structured to examine reproducibility across independent fabrication batches. In this context, the complementary compression and ring tensile tests were used not only to define construct mechanical properties, but also as quantitative indicators of manufacturing consistency. Preliminary observations also suggested reasonable mechanical stability up to 20 days under biologically relevant incubation conditions. The compressive modulus measured in this study (0.034 ± 0.01 MPa) is consistent with previously reported values for collagen-based hydrogels, which typically exhibit compressive moduli on the order of 0.01 MPa, depending on collagen concentration and processing conditions (Achilli and Mantovani, 2010). Likewise, the tensile Young’s modulus obtained from the ring tensile test (0.0358 ± 0.0031 MPa) falls within the range reported for engineered collagen-based vascular constructs (approximately 0.018–0.142 MPa), where the mechanical response strongly depends on the presence of embedded vascular smooth muscle cells or fibroblasts and on extracellular matrix remodeling (Seliktar et al., 2000; Berglund et al., 2005). In contrast, native arteries exhibit substantially higher stiffness. For example, the circumferential Young’s modulus of the human internal mammary artery has been reported to be approximately 8 MPa, while longitudinal values reach 16.8 MPa, reflecting the highly organized multilayered architecture and mature extracellular matrix of native vascular tissue (Stekelenburg et al., 2009; Henriques et al., 2023). Although the mechanical properties of the present constructs do not match those of native arteries, this outcome was anticipated. The primary objective of this work was not to reproduce the full biomechanical behavior of vascular tissue but rather to establish a reproducible FRESH bioprinting strategy capable of generating stable, geometrically accurate vascular constructs suitable for subsequent device-oriented and biological investigations. Achieving native arterial mechanics will likely require incorporating mechanically reinforcing biomaterials, advanced composite bioinks, prolonged tissue maturation, and/or cell-mediated extracellular matrix remodeling.

Beyond static mechanical profiling, perfusion and device-testing experiments demonstrated procedural compatibility while providing preliminary information on construct behavior under increasing flow conditions. Although the applied flow conditions were intentionally non-physiological, the observed pressure-dependent leakage highlights the intrinsic permeability of the hydrogel matrix, which may be advantageous in future studies focused on nutrient transport or endothelialization. Furthermore, stent deployment experiments demonstrated compatibility with intraluminal devices. The rigid polymeric structure accommodated local shape changes within the compliant hydrogel environment without abrupt construct failure, underscoring the utility of quantitative 3D imaging in evaluating vessel–device interaction. Importantly, perfusion and stent compatibility are not presented here as unique capabilities of the present platform. Ahmad et al. (2024), for example, demonstrated stent deployment and subsequent perfusion in an endothelialized coronary artery-sized bioprinted construct. In the present study, device deployment instead serves as a downstream procedural test of a construct whose fabrication-induced geometrical changes and batch-to-batch mechanical reproducibility had first been quantitatively characterized.

Biological experiments were intentionally designed as preliminary compatibility assessments. Our findings highlight a trade-off between fabrication robustness and biological permissiveness: while SA–GEL provided superior printability and geometrical reproducibility, collagen favored more physiologically relevant cell behavior, enhanced matrix interaction, and a more biologically permissive microenvironment (Hinton et al., 2015; Seliktar et al., 2000). Nonetheless, the results demonstrate that the proposed workflow supports sterile fabrication, cell viability up to 14 days, and initial endothelial adhesion, providing a controllable platform for future integration of biologically advanced bioinks and co-culture strategies.

To properly contextualize these outcomes, it is valuable to compare our integrated framework with representative vascular biofabrication strategies. Wang et al. (2022) fabricated small-caliber vascular conduits exhibiting physiological vasoactivity and suitability for surgical anastomosis, while Gold et al. (2021) developed biologically advanced models emphasizing multicellular organization and thromboinflammatory functionality. Portone et al. (2024) demonstrated a hybrid biofabricated vessel for intravascular medical-device testing and dynamic perfusion, whereas Ahmad et al. (2024) reported an endothelialized, coronary artery-sized bioprinted construct compatible with perfusion and intraluminal stent deployment. Within FRESH biofabrication, quantitative volumetric metrology has been demonstrated through OCT-based monitoring (Tashman et al., 2023), and Shiwarski et al. (2025) recently combined high-fidelity metrological characterization with internally perfusable FRESH architectures. Collectively, these studies show that the individual capabilities incorporated into our workflow—and, in some cases, combinations thereof—have already been demonstrated in advanced biofabricated systems.

Accordingly, the distinguishing contribution of the present study lies not in any individual technique, but in the integration of these techniques within a process-oriented framework centered on manufacturing reproducibility. Specifically, fabrication-induced geometric changes and mechanical reproducibility across independent batches are treated as quantitative manufacturing metrics before downstream evaluation of perfusion and intraluminal device compatibility. This positioning is particularly relevant to FRESH fabrication, where the geometrical freedom offered by embedded printing is accompanied by potential variability introduced during extraction and post-processing (Budharaju et al., 2024). In this context, our approach integrates micro-CT-based process monitoring, explicit batch-to-batch mechanical assessment, controlled perfusion, and intraluminal device deployment into a single process-oriented framework. By combining micro-CT-based process monitoring, batch-to-batch mechanical assessment, and subsequent procedural testing, the proposed workflow provides a deliberately simplified and quantitatively characterized framework complementary to biologically sophisticated vascular models.

Although this study focused on selected geometries, the proposed workflow is designed to be adaptable to different geometric configurations; however, this scalability requires further experimental validation. Direct comparison with rigid or elastomeric vascular phantoms represents an important future direction. Perfusion experiments were conducted under conservative steady-flow conditions to preserve construct integrity; consequently, wall shear stress values were likely below physiological levels. Nevertheless, the successful deployment and stable positioning of a stent-like ring provide a proof-of-concept foundation for future investigations under higher-flow and pulsatile regimes, enabling progressive evaluation of vessel–device interaction and imaging-guided assessment following validation of long-term construct stability.

Finally, transitioning to more biologically performant materials and conducting more comprehensive biological evaluations, including maturation of barrier function, chronic cell–matrix remodeling, and multicellular signaling, will be essential to further increase the platform’s physiological relevance. The engineering and metrological framework established here provides a quantitatively characterized foundation upon which these biological complexities can be progressively integrated.

## 5 Conclusion

This study presents a quantitatively characterized FRESH-bioprinting workflow for fabricating vessel-like constructs for device-oriented applications. Systematic optimization of bioink formulation, printing parameters, and post-processing conditions enabled reproducible fabrication across multiple geometrical scales. Non-destructive micro-CT provided a quantitative assessment of shape fidelity throughout the fabrication process, while complementary mechanical characterization demonstrated reproducible structural behavior and enabled evaluation of process-dependent variability. Preliminary biological and procedural investigations further demonstrate compatibility with sterile fabrication, cell viability up to 14 days, controlled perfusion, and intraluminal stent-like ring deployment.

Taken together, these findings establish a reproducible and quantitatively characterized experimental platform positioned between rigid vascular phantoms and biologically advanced tissue-engineered systems. The proposed workflow provides a robust experimental foundation for future process standardization, device-oriented vascular testing, and progressive biological refinement.

Future developments will include the integration of biologically advanced bioinks, dynamic and pulsatile perfusion, long-term biological maturation, barrier function, extracellular matrix remodeling, and expanded evaluation of interactions with endovascular devices. More broadly, the proposed workflow provides a foundation for the future development of standardized experimental benchmarks for the preclinical evaluation of endovascular technologies, thereby facilitating reproducibility and cross-laboratory comparison.

## Data Availability

The raw data supporting the conclusions of this article will be made available by the authors, without undue reservation.
