# Supplementary material for: FRESH-bioprinted vascular constructs: a quantitatively characterized workflow for reproducible device-oriented applications
Source: Front Bioeng Biotechnol. 2026 Sep 30;14:1920619. doi: 10.3389/fbioe.2026.1920619 (PMC13662051; doi:10.3389/fbioe.2026.1920619)
Supplement: Supplementary file 1 [file DataSheet1.docx]

**Table S1.** Confined compression test geometrical parameters.

| **Sample ID** | **D_ext_ (mm)** | **D_int_ (mm)** | **Platen separation (mm)** | **Cross-sectional area (mm^2^)** |
| --- | --- | --- | --- | --- |
| batch-1_1 | 9,99 | 6,87 | 5,32 | 41,35 |
| batch-1_2 | 8,98 | 7,21 | 7,13 | 22,51 |
| batch-1_3 | 8,72 | 6,88 | 5,96 | 22,46 |
| batch-1_4 | 9,32 | 7,03 | 6,38 | 29,41 |
| batch-1_5 | 8,64 | 7,11 | 6,87 | 18,93 |
| batch-2_1 | 9,51 | 7,52 | 8,68 | 26,6 |
| batch-2_2 | 8,95 | 7,04 | 7,52 | 23,97 |
| batch-2_3 | 8,8 | 7,11 | 8,6 | 21,04 |
| batch-2_4 | 9,3 | 6,96 | 8,55 | 29,8 |
| batch-2_5 | 10,24 | 8,18 | 8,51 | 29,77 |
| batch-3_1 | 9,01 | 7,05 | 8,43 | 24,64 |
| batch-3_2 | 9,74 | 6,64 | 9,59 | 39,86 |
| batch-3_3 | 9,85 | 6,54 | 9,52 | 42,56 |
| batch-3_4 | 9,72 | 7,21 | 10,22 | 33,41 |
| batch-3_5 | 9,64 | 7,14 | 10,02 | 32,95 |

**Table S2**. Ring tensile test geometrical parameters.

| **Sample ID** | **Width (mm)** | **Platen separation (mm)** | **Thickness (mm)** | **Cross-sectional area (mm^2^)** |
| --- | --- | --- | --- | --- |
| batch-1_1 | 6 | 10.72 | 1.38 | 16,56 |
| batch-1_2 | 7.22 | 9.12 | 1.27 | 18,34 |
| batch-1_3 | 6.54 | 10.15 | 1.785 | 23,35 |
| batch-1_4 | 7.13 | 10.12 | 1.34 | 19.11 |
| batch-1_5 | 7.06 | 9.98 | 1.29 | 18.21 |
| batch-2_1 | 8,85 | 9.38 | 2.095 | 37,08 |
| batch-2_2 | 8,67 | 10.77 | 1.22 | 21,15 |
| batch-2_3 | 8,08 | 9.51 | 2,42 | 39,11 |
| batch-2_4 | 8.74 | 10.01 | 1.98 | 34.61 |
| batch-2_5 | 8.23 | 9.96 | 2.05 | 33.74 |
| batch-3_1 | 8.65 | 8.92 | 1.67 | 28,89 |
| batch-3_2 | 8.4 | 9.61 | 1.807 | 20,35 |
| batch-3_3 | 8.43 | 9.23 | 1.177 | 19,84 |
| batch-3_4 | 8.54 | 9.41 | 1.73 | 29.55 |
| batch-3_5 | 8.51 | 9.33 | 1.62 | 27.57 |

**Figure S1. Micro-CT images of the printed model.** Micro-CT images of the printed model at 3% (left) and 6% (right) SA. Low-contrast 3% SA samples before bath melting could not be segmented due to overlapping greyscale histograms.

**Figure S2. Effect of incubation on mechanical stability.** Mechanical compression tests were performed on ring-shaped samples immediately after printing (T0) and after 20 days of incubation (T20). A slight decrease in mechanical stability was observed after 20 days, although this difference was not statistically significant.

**Figure S3. Experimental setup for perfusion testing of FRESH-bioprinted vascular constructs.** a) Custom-designed coupling module containing the bioprinted vessel-like construct connected to the perfusion tubing. b) Complete perfusion circuit used to evaluate construct integrity and procedural compatibility under flow, including a syringe pump, custom coupling module, and pressure sensors positioned upstream and downstream of the construct. The red inset provides a magnified view of the coupling module integrated into the circuit.
